# A Meta-Regression Analysis of the Effectiveness of Mosquito Nets for Malaria Control: The Value of Long-Lasting Insecticide Nets

**DOI:** 10.3390/ijerph15030546

**Published:** 2018-03-19

**Authors:** Gi-geun Yang, Dohyeong Kim, Anh Pham, Christopher John Paul

**Affiliations:** 1 Department of Fire Service Administration, Wonkwang University, 460 Iksan-daero, Iksan, Jeonbuk 570-749, Korea; withgg@wku.ac.kr; 2 School of Economic, Political and Policy Sciences, The University of Texas at Dallas, 800 W Campbell Road, Richardson, TX 75080, USA; 3 Department of Economics and International Business, Foreign Trade University, 91 Chua Lang, Dong Da, Hanoi 100000, Vietnam; Anh-thi-cam.Pham@utdallas.edu; 4 Department of Public Administration, North Carolina Central University, 1801 Fayetteville St., Durham, NC 27707, USA; cpaul5@nccu.edu

**Keywords:** malaria, mosquito nets, long-lasting insecticidal nets (LLINs), meta-regression

## Abstract

Long-lasting insecticidal nets (LLINs) have been widely used as an effective alternative to conventional insecticide-treated nets (ITNs) for over a decade. Due to the growing number of field trials and interventions reporting the effectiveness of LLINs in controlling malaria, there is a need to systematically review the literature on LLINs and ITNs to examine the relative effectiveness and characteristics of both insecticide nettings. A systematic review of over 2000 scholarly articles published since the year 2000 was conducted. The odds ratios (ORs) of insecticidal net effectiveness in reducing malaria were recorded. The final dataset included 26 articles for meta-regression analysis, with a sample size of 154 subgroup observations. While there is substantial heterogeneity in study characteristics and effect size, we found that the overall OR for reducing malaria by LLIN use was 0.44 (95% CI = 0.41–0.48, *p* < 0.01) indicating a risk reduction of 56%, while ITNs were slightly less effective with an OR of 0.59 (95% CI = 0.57–0.61, *p* <0.01). A meta-regression model confirms that LLINs are significantly more effective than ITNs in the prevention of malaria, when controlling for other covariates. For both types of nets, protective efficacy was greater in high transmission areas when nets were used for an extended period. However, cross-sectional studies may overestimate the effect of the nets. The results surprisingly suggest that nets are less effective in protecting children under the age of five, which may be due to differences in child behavior or inadequate coverage. Compared to a previous meta-analysis, insecticide-treated nets appear to have improved their efficacy despite the risks of insecticide resistance. These findings have practical implications for policymakers seeking effective malaria control strategies.

## 1. Introduction

With approximately 200 million cases per year, malaria is responsible for a large proportion of the global burden of disease and is a serious public health issue in Africa [1]. The fight against malaria has forged an extensive global public health initiative. Policymakers consider insecticide-treated nets (ITNs) one of the most successful vector control tools to combat malaria. Mosquito nets treated with synthetic pyrethroid insecticides have been shown to cause a decline in malaria morbidity and mortality in field efficacy trials carried out in different countries [2,3,4,5]. Over 427 million nets were delivered to households in Sub-Saharan Africa between 2012 and 2014 [1]. 

While the effectiveness of conventional ITNs in controlling malaria has been well established [4,6,7], there have been some operational problems hindering their long-term usefulness. The effective dose of insecticide on the ITN only lasts for a limited time. Thus, nets need to be re-treated regularly (at least once per year) [8]. However, regular re-treatment at the household or community level has proven very difficult to implement, resulting in low re-treatment rates. Moreover, repeatedly washing nets can reduces their protective efficacy [9]. In Africa, it is suggested that less than 5% of distributed ITNs were properly treated or re-treated [10]. A long-term study in Thailand reported that low re-treatment rates were a limiting factor in producing successful outcomes for an ITN intervention [11]. Despite notable efforts by many African countries and organizations (such as the Global Fund and the President’s Malaria Initiative) to reduce the incidence of malaria by increasing ITN coverage through mass distribution, the long-term effectiveness of ITNs for malaria control remains a concern.

As a simpler solution to the problems of conventional ITNs, long-lasting insecticide-treated nets (LLINs) have been developed using novel fabric technologies [12]. LLINs, recommended for malaria control by the World Health Organization, have insecticides either incorporated into or coated around their fibers. This resists multiple washes and remains effective for a longer time [13]. These long-lasting nets are designed to maintain their biological efficacy against vector mosquitoes for at least three years when used as recommended, eliminating the need for regular insecticide re-treatment. In terms of the cost per treated year of protection, LLINs are significantly cheaper to use than ITNs or indoor residual spraying (IRS). The annual cost for LLINs ranges from USD 1.48 to 2.64, as compared to IRS (USD 3.27 to 3.90 per year) and standard ITNs (USD 1.43 to 6.05 per year) [14,15,16]. Several trials also demonstrated the superior durability of LLINs over ITNs [17,18]. Compared with conventional ITNs that are treated by being dipped into insecticide, LLINs have several important advantages. This includes the fact that LLINs do not need to undergo re-treatment, their reduced insecticide consumption and their reduced insecticide release to the environment. 

Many field-based studies examining the efficacy of mosquito nets in reducing the incidence of malaria look at LLINs. However, most analyses combined all insecticide-treated nets, including both ITNs and LLINs. Some studies examined LLIN efficacy alone, but it is difficult to get a sense of large-scale performance of LLINs from individual studies. No study has assessed the overall effectiveness of LLINs in preventing malaria infection rates compared to ITNs. An early meta-analysis of ITN efficacy, published in 1995 by Choi et al., reported that ITNs reduced malaria cases by around 24% [6]. In his 2004 Cochrane review of insecticide-treated bed nets, Lengeler found a relative rate of malaria of 0.83 for children using ITNs compared to children who used no nets [4]. In many settings, LLINs ITNs are used in conjunction with IRS. There is only one published meta-analysis that has assessed the overall impact of IRS in reducing malaria cases [19]. No meta-regression study has examined any potential combined efficacy of using both IRS and LLINs. Fullman (2013) conducted the first multi-country analysis of the combined effectiveness of IRS and insecticidal netting, but only assessed children under the age of five, and the analysis combined both conventional ITNs and LLINs [20]. 

Meta-regression is a form of meta-analysis that has grown in popularity in recent years. This is in part due to the increasing numbers of systematic reviews and meta-analyses published in the biomedical literature [21]. Meta-regression is a statistical method of pooling covariate data from studies identified in a systematic quantitative analysis [22]. This method calculates the variation in results across studies and identifies the extent of variation caused by factors such as targeted groups, intervention types, study designs and measurement outcomes. This allows for the assessment of heterogeneity. We selected meta-regression as an appropriate approach to evaluate the effectiveness of LLINs and ITNs in malaria prevention under diverse conditions. In this paper, studies published since 2000 on ITNs or LLINs were systematically reviewed and data from suitable studies were consolidated using meta-regression analysis. The results are useful for malaria policymakers in assessing the efficacy of LLINs and ITNs, especially given the limited resources available to combat malaria.

## 2. Methods

### 2.1. Database Search and Systematic Review

Using five databases (PubMed, Google Scholar, Science Direct, JSTOR, and Malaria Journal) a comprehensive review of the literature published since 2000 was performed. Publications were searched for with keywords including malaria, long-lasting treated bed net, insecticide-treated bed net, LLIN, ITN, prevalence and incidence. This process identified 2051 articles. Experimental studies that reported on the protective efficacy of either LLINs or ITNs in human populations that adopted cross-sectional, cohort, or randomized controlled trial study designs were included. Articles were excluded if they met any of the following three criteria: they were review articles; they were articles reporting only the impact on mosquito populations; they were articles reporting ITN/LLIN interventions combined with untreated nets, IRS, or immunization. After this step, a total of 215 articles were identified for full review. The full texts of the 215 articles were read to confirm they qualified for inclusion in the meta-analysis. Articles were excluded that did not include sufficient numerical information for calculating the effectiveness of ITNs/LLINs, such as the numerical incidence of malaria before and after intervention (for cohort studies) or between treatment and control groups (for cross-sectional studies and randomized controlled trials). After this step, a total of 39 articles qualified for data collection and analysis.

### 2.2. Data Collection and Analysis

**Figure 1:**
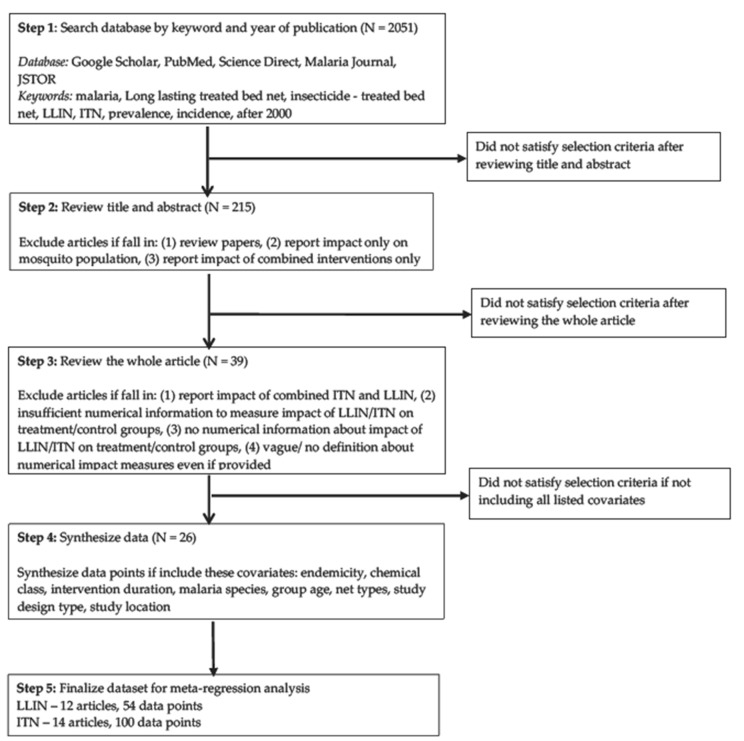
Methodology of searching for and selecting studies to include in meta-regression analysis.

Data from the 39 articles were recorded with quantitative measures for the following covariates: endemicity, chemical class, malaria species, intervention duration, group age, net types, study design and study location. The costs of treated nets were not recorded because almost all nets were supplied by social distribution campaigns, usually at a subsidized price. This approach promotes and distributes the socially beneficial intervention rather than commercializing the product. Thirteen articles were excluded due to a lack of quantitative data for at least one of the covariates listed above. The final sample for meta-analysis includes 54 data points from 12 LLIN (long-lasting insecticide-treated nets) articles and 100 data points from 14 ITN (insecticide-treated nets) articles. Figure 1 depicts the process used to search and select studies.

Odds ratios (ORs) were estimated for the effectiveness of treated nets. If the OR was not reported in a study, it was calculated based on malaria incidence before and after the intervention (cohort studies), or malaria incidence between the treatment and control groups (cross-sectional studies and randomized controlled trials). New variables were created from quantitative information synthesized from studies to enable a more comprehensive meta-regression analysis. Covariates for sample size, pesticide type, malaria parasite, location, study duration, study design, and participants under the age of five were incorporated into the model. Sample size was created by summing the total number of individuals in study sub-groups. Two dummy variables were created for the insecticides deltamethrin and alphapermethrin. These were compared with the insecticide permethrin as a baseline. A dummy variable was created for the type of malaria parasite, where 1 represents *Plasmodium falciparum* only, and 0 represents both *P. falciparum* and *Plasmodium vivax*. A dummy variable for the study location was created to assess the effectiveness of treated nets in Africa, the continent bearing the highest burden of malaria. To represent the studies undertaken in Africa, 1 was used, and 0 represented the studies undertaken in Asia. Due to a concern over the long-term durability of treated nets and the fact that ITNs need to be retreated at least once a year to ensure continued insecticidal effectiveness, a dummy variable was created for the duration of the study, where 1 represented a study duration longer than 12 months and 0 represented a study duration of less than one year. Additionally, two dummy variables were created to indicate cohort and cross-sectional study designs, compared with randomized controlled trials as baseline. A dummy variable was created for children under the age of five to investigate the marginal impact of treated nets on this vulnerable group [1,23]. There were insufficient studies to create a dummy variable for pregnant women (another vulnerable population). An endemicity covariate was not necessary because almost all studied areas were reported as hyperendemic for malaria, except one LLIN [24] and two ITN studies [25,26] that did not report endemicity. Finally, and most importantly, net type is a dummy variable where 1 represents LLIN only and 0 represents ITN only. 

To conduct a meta-analysis of the odds of malaria prevalence, we implemented a regression meta-analysis using the statistical package Stata (StataCorp. 2017. Stata Statistical Software: Release 15. College Station, TX: StataCorp LLC). The adjusted R-Square value for the regression is calculated in the statistical software following the formula of the unadjusted R-Square multiplied by a ratio of the degrees of freedom to the residual degrees of freedom.

## 3. Results

### 3.1 Description of Articles Included in the Final Data

Of the 14 ITN articles, six were randomized controlled trials [3,5,7,27,28,29], six were cross-sectional studies [2,25,26,30,31,32] that compared malaria prevalence between a group that slept under ITNs and a group that did not and two were cohort studies [33,34] that analyzed malaria rates within groups before and after using an ITN. Twelve articles addressed malaria caused by *P. falciparum* or *P. vivax*, and two articles did not note the species [2,3]. Of the 12 ITN articles identifying the species of malaria, eight articles addressed *P. falciparum* only [25,26,27,30,31,32,33,34] while the remaining four articles reported for both species [5,7,28,29], in which *P. falciparum* accounted for about 80–90% of total infections. Of the 14 ITN articles, three were conducted within Asia [5,28,29] (82 observations representing 82% of the ITN data) and 11 trials examined African countries [2,3,7,25,26,27,30,31,32,33,34] (18 observations representing 18% of the ITN data). The mean study size was 1500 participants. Of all studies, 13% of observations were in children under five years of age [2,3,7,25,26,27,30,32,34]. Two studies focused on pregnant women from 15 to 39 years of age [31,33], which accounted for 4% of the total data. 

Of the 12 LLIN articles, five were randomized controlled trials [35,36,37,38,39], four were cross-sectional studies [24,40,41,42], and three were cohort studies [9,43,44]. Among these 12 articles, seven articles reported *P. falciparum* only [24,38,39,40,41,42,43], four articles reported malaria caused by both *P. falciparum* and *P. vivax* [9,35,36,44], and one article did not mention the vector species [37]. Seven studies reported a protective efficacy for LLINs in Asia [9,24,35,36,37,39,44] (33 observations representing 61% of the LLIN data) and five studies had study locations in African countries [38,40,41,42,43] (21 observations representing 39% of the LLIN data). The mean study size was 1728 participants. A total of 37% of LLIN observations looked at children under five years of age [40,41,42,43]. None of the 12 studies addressed a population of pregnant women. A summary of ITN and LLIN article covariates is shown in Table 1.

### 3.2. Meta-Regression Analysis

**Figure 2:**
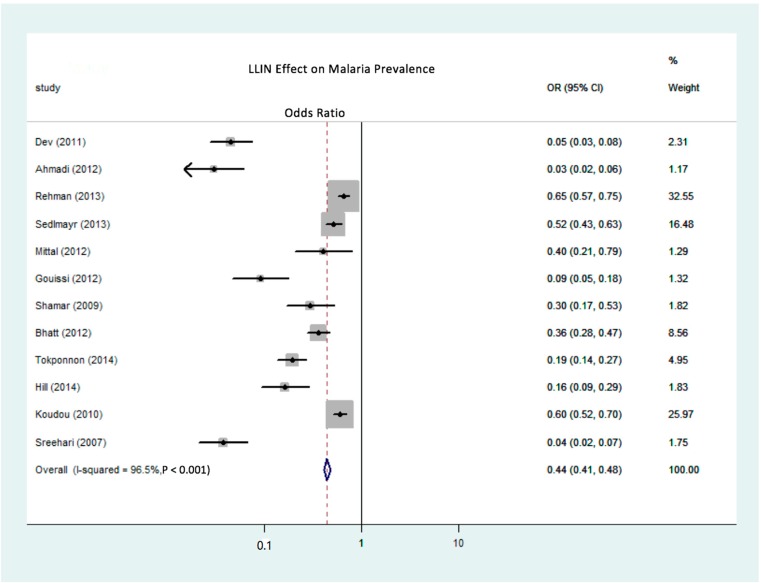
Forest plot of the meta-regression analysis of the odds ratio of the reduction of malaria prevalence due to the use of LLINs. (The row-like symbol for Ahmadi (2012) indicates that the lower limit of 95% CI for the study is so low that it goes beyond the chart area.).

**Figure 3:**
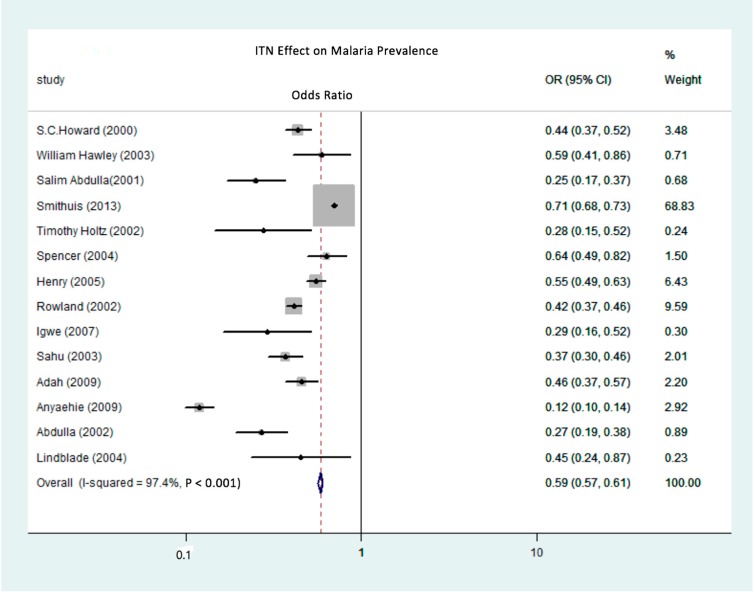
Forest plot of the meta-regression analysis of the odds ratios of the reduction of malaria prevalence due to the use of ITNs.

The effectiveness of treated nets in reducing the prevalence of malaria is recorded as an odds ratio (OR), representing the relative odds of contracting malaria between a treatment group and a control group. Studies were subdivided into two groups for analysis based on the type of net, either ITN or LLIN. Figure 2 depicts the OR between the LLIN treatment and without (or before) an LLIN treatment, while Figure 3 depicts a comparison of the OR for the contraction of malaria between ITN users and ITN non-users. The overall summary OR for LLIN effectiveness in reducing the prevalence of malaria is 0.44. This statistically significant finding indicates that the odds of someone in the LLIN users group contracting malaria were 56% less than in the LLIN non-users group, with the true population effect lying between 52% and 59% (*p* = 0.0001). Figure 2 also shows the ITN effectiveness summary OR is 0.59, indicating the odds of contracting malaria in the ITN group were 41% less than in the group without any ITN intervention, with the true population effect between 39% and 43% (*p* = 0.0001). This meta-regression analysis reveals that the efficacy of both ITNs and LLINs in reducing the prevalence of malaria is strong and statistically significant (*p* = 0.0001). This study also suggests that LLINs are more effective than ITNs in reducing the prevalence of malaria (as indicated in the meta-regression result for the LLIN covariate, shown in Table 2).

Even though a meta-regression analysis revealed that LLINs have a greater protective efficacy than ITNs, there remains a high level of heterogeneity between individual studies. As shown in Figure 2 and Figure 3, using 154 observations from both sub-groups, the meta-regression model identifies sources of heterogeneity such as target group characteristics, study conditions, and study designs. In the LLIN sub-group, the largest reported effect was found in the study by Ahmadi (2012) [44], in which the OR for the protective benefit of using LLINs was 0.03, which represented a 97% decrease in malaria prevalence in the LLIN user group compared to the LLIN non-user group. Meanwhile, the LLIN OR was approximately 0.65 in the study conducted by Rehman et al. (2013) [41]. ITN treatments have shown a similar range: ORs from 0.12 [33] to 0.71 [29] have been reported. 

In the meta-regression model, the dependent variable is the log transformation of the OR for net usage in regard to malaria prevention. The primary independent variables are whether an LLIN was used, the log-transformed proportion of study population with malaria before the intervention, the total sample size, whether the study duration lasted longer than 12 months, children under the age of five, the use of deltamethrin and alphapermethrin, *P. falciparum* only, and cohort and cross-sectional study designs. An OR of significantly less than 1 or a negative log OR implies that an increase in a covariate is associated with a decrease in the risk of contracting malaria. Use of LLINs, the log transformation of the initial prevalence prior to intervention, whether the study lasted longer than 12 months and whether children less than five years of age were present in the study are variables that were hypothesized to be negatively related to the OR. A model using the African covariate had a lower adjusted R-square, as well as an insignificant coefficient for Africa. Therefore, the African covariate was not included in the final model. The best-fit model is shown in Table 2.

As hypothesized, LLIN usage, initial malaria prevalence, and the study duration were shown to be strong and statistically significant negative predictors of the OR for malaria prevalence. Overall, the meta-regression results can be interpreted as follows: Firstly, the negative relationship of initial malaria prevalence to the log transformation of the OR indicated that the use of LLIN/ITN confers a stronger protection against contracting malaria for people living in malaria-endemic areas. Second, the negative relationship of the log transformation of the OR to whether the study lasted longer than 12 months implies that insecticidal nets were effective over longer periods of time if they are either long-lasting or routinely re-treated. As noted, conventional ITNs needs to be re-treated at least once a year to maintain insecticidal effectiveness. Third, contrary to expectation, the coefficient for children under the age of five was positive and significant, indicating that treated nets are less effective in preventing malaria for this age group compared to children aged five years or over. However, nets were somewhat effective in reducing malaria prevalence in both age categories. Fourth, cross-sectional study design was negatively related to the OR, as compared to cohort study and randomized controlled trial study designs, which could suggest that cross-sectional studies overestimated the effect of mosquito nets [45]. Fifth, according to the meta-regression results, the dummy variable of LLIN usage had a statistically significant effect on the log transformation of the OR when controlling for other variables. This indicates that LLIN usage confers greater protection against malarial infection than ITN usage. Finally, the analysis controlled for any effect of total sample size, different malaria types (*P. falciparum* and *P. vivax*), and different insecticides (deltamethrin, alphapermethrin and permethrin). There was no difference in net efficacy for these covariates.

## 4. Discussion

In the meta-analysis study published Choi et al., ITNs were shown to avert around 24% of malaria cases [6]. The present study, using only literature since the year 2000, provides a comparison with findings in 1995. This meta-analysis demonstrates how much ITN efficacy has improved as well as examining the novel technologies and materials of LLINs. This meta-regression finds that the overall effectiveness of ITNs and LLINs in reducing the incidence of malaria is 41% and 56% respectively, when compared to people who used no form of netting. This implies that the efficacy of treated nets has improved substantially over the last decade, despite concerns that pyrethroid resistance in mosquitoes may compromise the ability of treated nets to kill mosquitoes.

There are some differences between Choi et al.’s study and the present study. In the 1995 study, eight of the ten total field trials included in the analysis limited the study population to children less than 15 years old. The other two studies included in Choi et al.’s analysis contained both children and adults. Choi et al.’s analysis used two sub-groups based on the type of control. These were an ITN intervention group versus a group that received an untreated bed net, and a group that received permethrin-impregnated bed nets as compared to a group that received no net. Each sub-group used small samples of six field trials, comparing measures of the incidence of parasitemia as a function of person-weeks at risk for parasitemia. In this analysis, the sample study size increased to 26 studies (published since the year 2000), in which 14 studies reported on the effectiveness of ITNs and 12 reported on the effectiveness of LLINs. This analysis focused on the protective efficacy of both LLINs and ITNs and their protective efficacy when used in conjunction with each other. This study used the malaria prevalence odds ratio as the main indicator of infection prevention. In this analysis and unlike Lengeler’s [4], the target population is not limited to children, and includes analysis from the entire study population at risk.

Meta-analyses, such as the one presented here, are particularly useful for policymakers in strategically allocating resources in disease control. The main finding of this meta-analysis is the outstanding protective efficacy of LLINs over ITNs over long periods of time. LLINs are designed so that their insecticidal activity could last as long as the nets themselves, a claim which is supported by this meta-analysis. An additional finding is that even though some studies show that vulnerable groups (infants, young children, and pregnant women) sleep under insecticidal bed netting more often than others [46,47], the meta-regression results find that insecticide nets do not confer a higher protective efficacy for children under the age of five. Even though in regard to the distribution of interventions, priority is given to the most vulnerable, children of varying ages often sleep together. Nets are not designed to cover multiple children and thus, coverage is often inadequate. Besides, nets may be uncomfortable to use in limited spaces and hot, humid climates, which can result in reduced use of bed nets [48]. As infants and young children often sleep with their mothers, adult sleeping preferences can affect children’s exposure. During the dry season in many places, it is common for people to sleep unprotected for at least half the night [49,50]. Additionally, evidence suggests that the possession and appropriate use of treated netting does not automatically mean that these nets are used correctly [51,52]. The frequent perception of malaria as a common disease leads people to believe that it is not worth the inconvenience of daily net use. Recipients have even re-sold donated bed nets. These reasons are consistent with the disheartening report that in 2005 in Africa, it was estimated that only 3% of children under the age of five slept under insecticide-treated nets [26,53]. A recent study reports that only 14% of ITNs were used by children under the age of five [52], which is consistent with the known barriers to the correct use of ITNs mentioned above. To increase motivation for malaria prevention, populations in endemic areas need to be well educated about the severity of malaria and the value of bed netting as an infection prevention strategy. This requires better communication between authorities, social scientists, health professionals and target populations. Moreover, more efficient community-based strategies to prevent malaria infection are desperately needed in high endemic and rural areas [26]. 

The limitations of this study are common to meta-analyses. A meta-analysis of the literature only examines published studies. The comprehensive effectiveness of LLINs and ITNs might not be captured if most published studies focus on success stories, while insignificant impacts are not published. Several studies were excluded due to missing selection criteria for the adopted meta-regression model, which may have led to bias. Also, the studies used in the meta-analysis vary by approach. For example, most reported that the distribution of netting was free, though some studies involved the promotion and/or marketing of nets. The price of nets that users have to pay may affect proper use and deployment [54]. More broadly, there is substantial heterogeneity in the parameter measurements of the included studies. It is therefore difficult to determine to which populations we can apply our findings. Various subgroup analyses based on study designs, study conditions or target group characteristics could further explore the critical sources of heterogeneity. Furthermore, many studies failed to document coverage rates, even though high coverage rates are needed to realize the full potential of treated nets. In other words, the finding is based on an “intention to treat” analysis—an analysis of people who could be covered by the distributed nets, not an analysis of people who are confirmed to be using the nets. Therefore, potential effectiveness could be underestimated due to an assumption that all household members slept under the distributed nets. Finally, the meta-regression model R-square of 33% is not high and the sample size of 154 data points is quite small. However, a finding based on an “intention to treat” as compared to the true rate would result in a lower R-square. Small sample size is a common challenge for meta-regression studies [6,19]. Moreover, the performance of treated nets can vary depending on the setting and conditions. 

There remains high global demand for sustainable malaria control and elimination. Provided that people use treated nets properly and consistently, LLINs and ITNs are highly effective at preventing malaria [1,4]. Replacing ITNs with LLINs and achieving universal access to LLINs would greatly advance malaria control for all populations [55]. Many millions of conventional ITNs remain in routine use. However, it is important to balance the equity of maintaining widespread ITN usage while also making a more intensified effort to replace ITNs with LLINs.

## 5. Conclusions

The meta-regression model presented in this paper confirms that LLINs are statistically more effective than ITNs in preventing malaria, although the effectiveness of both has been substantially improved during the past decade. The findings support the importance of treated nets, and the improvement from LLINs, in malaria control.

## Figures and Tables

**Table 1:** Study descriptions (counts of article characteristics).

Covariates		ITN	LLIN
	Total Articles	14	12
**Study types**	Randomized Control Trials	6	5
	Cross-sectional	6	4
	Cohort	2	3
**Study locations**	Africa	11	5
	Asia	3	7
**Malaria species**	*P. falciparum* only	8	7
	*P. falciparum* and *P. vivax*	4	4
**Chemical class**	Permethrin	6	5
	Deltamethrin	5	5
	Alphapermethrin	1	2
**Age**	Children under 5	9	4

**Table 2:** Meta-regression results (*n* = 154) of a reduction in malaria prevalence by LLIN/ITN.

Covariate (Dependent Variable = log OR)	Coefficient (95% Confidence Interval)
Use of LLIN (only)	−0.954 (−1.419 to −0.49) ***
Log of initial prevalence (malaria proportion before intervention)	−0.224 (−0.355 to −0.092) ***
Total sample size (thousands)	−0.0066 (−0.076 to 0.062)
Study duration (more than 12 months)	−0.451 (−0.868 to −0.034) **
Use of deltamethrin	0.228 (−0.195 to 0.653)
Use of alphapermethrin	0.399 (−0.263 to 1.063)
Children under the age of five	1.313 (0.821 to 1.804) ***
*Plasmodium falciparum* (only)	−0.274 (−0.661 to 0.112)
Cohort study design	−0.336 (−0.845 to 0.171)
Cross-sectional study design	−0.7 (−1.301 to −0.099 ) **
Constant	−0.980 (−1.594 to −0.367) ***
Adjusted R-square	0.33

Note: ** *p* < 0.05, *** *p* < 0.01.

## References

[1] World Health Organization (WHO) (2016). World Malaria Report 2016.

[2] Abdulla S., Schellenberg J.A., Nathan R., Mukasa O., Marchant T., Smith T., Tanner M., Lengeler C. (2001). Impact on malaria morbidity of a programme supplying insecticide treated nets in children aged under 2 years in Tanzania: Community cross sectional study. BMJ.

[3] Howard S.C., Omumbo J., Nevill C., Some E.S., Donnelly C.A., Snow R.W. (2000). Evidence for a mass community effect of insecticide-treated bednets on the incidence of malaria on the Kenyan coast. Trans. R. Soc. Trop. Med. Hyg..

[4] Lengeler C. (2004). Insecticide-treated bed nets and curtains for preventing malaria. Cochrane Database Syst. Rev..

[5] Rowland M., Webster J., Saleh P., Chandramohan D., Freeman T., Pearcy B., Durrani N., Rab A., Mohammed N. (2002). Prevention of malaria in Afghanistan through social marketing of insecticide-treated nets: Evaluation of coverage and effectiveness by cross-sectional surveys and passive surveillance. Trop. Med. Int. Health.

[6] Choi H.W., Breman J.G., Teutsch S.M., Liu S., Hightower A.W., Sexton J.D. (1995). The effectiveness of insecticide-impregnated bednets in reducing cases of malaria infection: A meta-analysis of published results. Am. J. Trop. Med. Hyg..

[7] Hawley W.A., Phillips-Howard P.A., ter Kuile F.O., Terlouw D.J., Vulule J.M., Ombok M., Nahlen B.L., Gimnig J.E., Kariuki S.K., Kolczak M.S. (2003). Community-wide effects of permethrin-treated bed nets on child mortality and malaria morbidity in western Kenya. Am. J. Trop. Med. Hyg..

[8] World Health Organization (WHO) (2002). Instructions for Treatment and Use of Insecticide-Treated Mosquito Nets.

[9] Dev V., Phookan S., Padhan K., Tewari G.G., Khound K. (2011). Laboratory wash-resistance and field evaluation of deltamethrin incorporated long-lasting polyethylene netting (Netprotect (R)) against malaria transmission in Assam, north-east India. Acta Trop..

[10] Dabire R.K., Diabate A., Baldet T., Pare-Toe L., Guiguemde R.T., Ouedraogo J.B., Skovmand O. (2006). Personal protection of long lasting insecticide-treated nets in areas of Anopheles gambiae s.s. resistance to pyrethroids. Malar. J..

[11] Patipong S., Yongchaitrakul S. (2008). Field efficacy and persistence of Long Lasting Insecticide treated mosquito Nets (LLINs) in comparison with conventional Insecticide Treated mosquito Nets (ITN) against malaria vector in Thailand. J. Vector Borne Dis..

[12] Guillet P., Alnwick D., Cham M.K., Neira M., Zaim M., Heymann D., Mukelabai K. (2001). Long-lasting treated mosquito nets: A breakthrough in malaria prevention. Bull. World Health Organ..

[13] World Health Organization (WHO) (2014). WHO Recommendations for Achieving Universal Coverage with Long-Lasting Insecticidal Nets in Malaria Control.

[14] Yukich J., Tediosi F., Lengeler C. (2007). Comparative Cost-Effectiveness of ITNs or IRS in Sub-Saharan Africa.

[15] Programme G.M. (2007). Insecticide-Treated Mosquito Nets: A WHO Position Statement.

[16] Yukich J.O., Lengeler C., Tediosi F., Brown N., Mulligan J.A., Chavasse D., Stevens W., Justino J., Conteh L., Maharaj R. (2008). Costs and consequences of large-scale vector control for malaria. Malar. J..

[17] Graham K., Kayedi M.H., Maxwell C., Kaur H., Rehman H., Malima R., Curtis C.F., Lines J.D., Rowland M.W. (2005). Multi-country field trials comparing wash-resistance of PermaNet and conventional insecticide-treated nets against anopheline and culicine mosquitoes. Med. Vet. Entomol..

[18] Sreehari U., Raghavendra K., Rizvi M.M., Dash A.P. (2009). Wash resistance and efficacy of three long-lasting insecticidal nets assessed from bioassays on Anopheles culicifacies and Anopheles stephensi. Trop. Med. Int. Health.

[19] Kim D., Fedak K., Kramer R. (2012). Reduction of malaria prevalence by indoor residual spraying: A meta-regression analysis. Am. J. Trop. Med. Hyg..

[20] Fullman N., Burstein R., Lim S.S., Medlin C., Gakidou E. (2013). Nets, spray or both? The effectiveness of insecticide-treated nets and indoor residual spraying in reducing malaria morbidity and child mortality in sub-Saharan Africa. Malar. J..

[21] Haidich A.B. (2010). Meta-analysis in medical research. Hippokratia.

[22] Thompson S.G., Higgins J. (2002). How should meta-regression analyses be undertaken and interpreted?. Stat. Med..

[23] World Health Organization (WHO) (2017). Children: Reducing Mortality.

[24] Sreehari U., Razdan R.K., Mittal P.K., Ansari M.A., Rizvi M.M., Dash A.P. (2007). Impact of Olyset nets on malaria transmission in India. J. Vector Borne Dis..

[25] Adah P.O., Mafiana C.F., Sam-Wobo S.O. (2009). Impact assessment of the use of insecticide-treated bed nets on parasitaemia and anaemia for malaria control in children, Ogun State, Nigeria. Public Health.

[26] Holtz T.H., Marum L.H., Mkandala C., Chizani N., Roberts J.M., Macheso A., Parise M.E., Kachur S.P. (2002). Insecticide-treated bednet use, anaemia, and malaria parasitaemia in Blantyre District, Malawi. Trop. Med. Int. Health.

[27] Lindblade K.A., Eisele T.P., Gimnig J.E., Alaii J.A., Odhiambo F., ter Kuile F.O., Hawley W.A., Wannemuehler K.A., Phillips-Howard P.A., Rosen D.H. (2004). Sustainability of reductions in malaria transmission and infant mortality in western Kenya with use of insecticide-treated bednets: 4 to 6 years of follow-up. J. Am. Med. Assoc..

[28] Sahu S.S., Jambulingam P., Vijayakumar T., Subramanian S., Kalyanasundaram M. (2003). Impact of alphacypermethrin treated bed nets on malaria in villages of Malkangiri district, Orissa, India. Acta Trop..

[29] Smithuis F.M., Kyaw M.K., Phe U.O., van der Broek I., Katterman N., Rogers C., Almeida P., Kager P.A., Stepniewska K., Lubell Y. (2013). The effect of insecticide-treated bed nets on the incidence and prevalence of malaria in children in an area of unstable seasonal transmission in western Myanmar. Malar. J..

[30] Abdulla S., Schellenberg J.R., Mukasa O., Lengeler C. (2002). Usefulness of a dispensary-based case-control study for assessing morbidity impact of a treated net programme. Int. J. Epidemiol..

[31] Igwe P.C., Inem V., Ebuehi O.M., Afolabi B.M. (2007). The effect of insecticide treated bed net use on malaria episodes, parasitaemia and haemoglobin concentration among primigravidae in a peri-urban settlement in southeast Nigeria. J. Rural Trop. Public Health.

[32] Spencer S., Grant A.D., Piola P., Tukpo K., Okia M., Garcia M., Salignon P., Genevier C., Kiguli J., Guthmann J.P. (2004). Malaria in camps for internally-displaced persons in Uganda: Evaluation of an insecticide-treated bednet distribution programme. Trans. R. Soc. Trop. Med. Hyg..

[33] Anyaehie U., Nwagha U.I., Aniebue P.N., Nwagha T.U. (2011). The effect of free distribution of insecticide-treated nets on asymptomatic Plasmodium parasitemia in pregnant and nursing mothers in a rural Nigerian community. Niger. J. Clin. Pract..

[34] Henry M.C., Assi S.B., Rogier C., Dossou-Yovo J., Chandre F., Guillet P., Carnevale P. (2005). Protective efficacy of lambda-cyhalothrin treated nets in Anopheles gambiae pyrethroid resistance areas of Cote d'Ivoire. Am. J. Trop. Med. Hyg..

[35] Bhatt R.M., Sharma S.N., Uragayala S., Dash A.P., Kamaraju R. (2012). Effectiveness and durability of Interceptor(R) long-lasting insecticidal nets in a malaria endemic area of central India. Malar. J..

[36] Hill N., Zhou H.N., Wang P., Guo X., Carneiro I., Moore S.J. (2014). A household randomized, controlled trial of the efficacy of 0.03% transfluthrin coils alone and in combination with long-lasting insecticidal nets on the incidence of Plasmodium falciparum and Plasmodium vivax malaria in Western Yunnan Province, China. Malar. J..

[37] Mittal P.K., Sood R.D., Kapoor N., Razdan R.K., Dash A.P. (2012). Field evaluation of Icon(R)Life, a long-lasting insecticidal net (LLIN) against Anopheles culicifacies and transmission of malaria in District Gautam Budh Nagar (Uttar Pradesh), India. J. Vector Borne Dis..

[38] Sedlmayr R., Fink G., Miller J.M., Earle D., Steketee R.W. (2013). Health impact and cost-effectiveness of a private sector bed net distribution: Experimental evidence from Zambia. Malar. J..

[39] Sharma S.K., Tyagi P.K., Upadhyay A.K., Haque M.A., Mohanty S.S., Raghavendra K., Dash A.P. (2009). Efficacy of permethrin treated long-lasting insecticidal nets on malaria transmission and observations on the perceived side effects, collateral benefits and human safety in a hyperendemic tribal area of Orissa, India. Acta Trop..

[40] Modeste Gouissi F., Salifou S., Patrick Edorh A., Anges Yadouleton W., Djenontin A., Bio-Banganna S., Geoffroy Augustin Gouissi S., Akogbeto M. (2012). Assessment of Long-Lasting Insecticidal Nets (LLINs) on Vectors and Malaria Transmission in the Commune of Aguegues, Benin. BioImpacts.

[41] Rehman A.M., Mann A.G., Schwabe C., Reddy M.R., Roncon Gomes I., Slotman M.A., Yellott L., Matias A., Caccone A., Nseng Nchama G. (2013). Five years of malaria control in the continental region, Equatorial Guinea. Malar. J..

[42] Tokponnon F.T., Ogouyemi A.H., Sissinto Y., Sovi A., Gnanguenon V., Cornelie S., Adeothy A.A., Osse R., Wakpo A., Gbenou D. (2014). Impact of long-lasting, insecticidal nets on anaemia and prevalence of Plasmodium falciparum among children under five years in areas with highly resistant malaria vectors. Malar. J..

[43] Koudou B.G., Ghattas H., Esse C., Nsanzabana C., Rohner F., Utzinger J., Faragher B.E., Tschannen A.B. (2010). The use of insecticide-treated nets for reducing malaria morbidity among children aged 6–59 months, in an area of high malaria transmission in central Cote d’Ivoire. Parasit. Vectors.

[44] Soleimani-Ahmadi M., Vatandoost H., Shaeghi M., Raeisi A., Abedi F., Eshraghian M.R., Madani A., Safari R., Oshaghi M.A., Abtahi M. (2012). Field evaluation of permethrin long-lasting insecticide treated nets (Olyset (R)) for malaria control in an endemic area, southeast of Iran. Acta Trop..

[45] Jadad A.R., Enkin M.W. (2007). Bias in Randomized Controlled Trials. Randomized Controlled Trials: Questions, Answers, and Musings.

[46] Lam Y., Harvey S.A., Monroe A., Muhangi D., Loll D., Kabali A.T., Weber R. (2014). Decision-making on intra-household allocation of bed nets in Uganda: Do households prioritize the most vulnerable members?. Malar. J..

[47] Ricotta E., Koenker H., Kilian A., Lynch M. (2014). Are pregnant women prioritized for bed nets? An assessment using survey data from 10 African countries. Glob. Health Sci. Pract..

[48] Toe L.P., Skovmand O., Dabire K.R., Diabate A., Diallo Y., Guiguemde T.R., Doannio J.M., Akogbeto M., Baldet T., Gruenais M.E. (2009). Decreased motivation in the use of insecticide-treated nets in a malaria endemic area in Burkina Faso. Malar. J..

[49] Frey C., Traore C., De Allegri M., Kouyate B., Muller O. (2006). Compliance of young children with ITN protection in rural Burkina Faso. Malar. J..

[50] Nuwaha F. (2002). People’s perception of malaria in Mbarara, Uganda. Trop. Med. Int. Health.

[51] Ye Y., Patton E., Kilian A., Dovey S., Eckert E. (2012). Can universal insecticide-treated net campaigns achieve equity in coverage and use? the case of northern Nigeria. Malar. J..

[52] Zollner C., De Allegri M., Louis V.R., Ye M., Sie A., Tiendrebeogo J., Jahn A., Muller O. (2015). Insecticide-treated mosquito nets in rural Burkina Faso: Assessment of coverage and equity in the wake of a universal distribution campaign. Health Policy Plan.

[53] Roll Back Malaria-World Health Organization (RBM-WHO) (2005). World Malaria Report.

[54] Tarozzi A., Mahajan A., Blackburn B., Kopf D., Krishnan L., Yoong J. (2014). Micro-Loans, Insecticide-Treated Bednets, and Malaria: Evidence from a Randomized Controlled Trial in Orissa, India. Am. Econ. Rev..

[55] Koenker H., Kilian A. (2014). Recalculating the Net Use Gap: A Multi-Country Comparison of ITN Use versus ITN Access. PLoS ONE.

